# Strategies for robust, accurate, and generalizable benchmarking of drug discovery platforms

**DOI:** 10.1101/2024.12.10.627863

**Published:** 2024-12-16

**Authors:** Melissa Van Norden, William Mangione, Zackary Falls, Ram Samudrala

**Affiliations:** 1 Department of Biomedical Informatics, Jacobs School of Medicine and Biomedical Sciences, University at Buffalo, State University of New York, Buffalo, NY, USA.

## Abstract

Benchmarking is an important step in the improvement, assessment, and comparison of the performance of drug discovery platforms and technologies. We revised the existing benchmarking protocols in our Computational Analysis of Novel Drug Opportunities (CANDO) multiscale therapeutic discovery platform to improve utility and performance. We optimized multiple parameters used in drug candidate prediction and assessment with these updated benchmarking protocols. CANDO ranked 7.4% of known drugs in the top 10 compounds for their respective diseases/indications based on drug-indication associations/mappings obtained from the Comparative Toxicogenomics Database (CTD) using these optimized parameters. This increased to 12.1% when drug-indication mappings were obtained from the Therapeutic Targets Database. Performance on an indication was weakly correlated (Spearman correlation coefficient >0.3) with indication size (number of drugs associated with an indication) and moderately correlated (correlation coefficient >0.5) with compound chemical similarity. There was also moderate correlation between our new and original benchmarking protocols when assessing performance per indication using each protocol. Benchmarking results were also dependent on the source of the drug-indication mapping used: a higher proportion of indication-associated drugs were recalled in the top 100 compounds when using the Therapeutic Targets Database (TTD), which only includes FDA-approved drug-indication associations (in contrast to the CTD, which includes associations drawn from the literature). We also created compbench, a publicly available head-to-head benchmarking protocol that allows consistent assessment and comparison of different drug discovery platforms. Using this protocol, we compared two pipelines for drug repurposing within CANDO; our primary pipeline outperformed another similarity-based pipeline still in development that clusters signatures based on their associated Gene Ontology terms. Our study sets a precedent for the complete, comprehensive, and comparable benchmarking of drug discovery platforms, resulting in more accurate drug candidate predictions.

## 2 Introduction

Drug discovery is a difficult problem: according to one 2010 estimate, 24.3 early “target-to-hit” projects were completed per approved drug. [1] These preclinical projects were estimated to account for at least 31% and up to 43% of total drug discovery expenditure. [1, 2] The result is a high and increasing price for novel drug development, with estimates ranging from $985 million to over $2 billion for one new drug to be successfully brought to market. [2-4] The creation and refinement of more effective computational drug discovery pipelines promises to reduce the failure rate and increase the cost-effectiveness of drug discovery [5, 6]. This is already an active field, with thousands of articles published and multiple drugs discovered and/or optimized through computational methods already in use. [7, 8] Modern drug discovery and repurposing techniques range from traditional single-target molecular docking and retrospective clinical analysis to more novel signature matching, network/pathway mapping, and deep learning platforms. [9-11] The successes and failures of novel and repurposed therapeutics in fighting the rapid rise and spread of COVID-19 made more clear than ever that robust and effective drug discovery pipelines are essential for healthcare in a modern world. [11-14] Still, systems for the assessment, incorporation, and adoption of the discoveries of computational platforms and studies need improvement and standardization [15].

For this study, we define a drug discovery platform as consisting of one or more pipelines, themselves comprising protocols (such target selection, docking, interaction scoring, and/or compound ranking), that come together to allow the prediction of novel drug candidates for one or more indications. This excludes platforms that facilitate drug discovery but do not, themselves, predict novel drug-indication associations, such as those for drug-target interaction prediction. Benchmarking is the process of assessing and comparing the practical utility of existing platforms, pipelines, and protocols. [16, 17] In drug discovery, quality benchmarking can assist in (1) designing, refining, and optimizing computational pipelines; (2) estimating performance on novel drug candidate predictions; and (3) choosing the most suitable pipeline for a specific scenario (e.g., repurposing a drug for a novel disease/indication). Neutral studies that impartially compare multiple indications and protocols are the gold standard in benchmarking [16-18] However, such studies are both more difficult to complete and less prioritized by high-ranking journals than those reporting novel methods. [17, 19] Differing ground truth data, metrics, and benchmarking protocols render benchmarking results incomparable between studies of individual pipelines. [15, 20] Authors may compare their drug discovery pipelines to others, but these comparisons are generally restricted to similar pipelines that use similar input data. [21-54] Head-to-head benchmarking also tends to find the authors’ pipeline superior due to publication bias, greater familiarity with one’s own protocols, selective metric reporting, information leak, and overfitting. [19, 55] This makes benchmarking less useful for developers, end users, and the scientific community as a whole in determining which drug discovery pipelines perform how well under what circumstances. [17, 19]

Current drug discovery benchmarking protocols vary widely from study to study. [15, 20] Drug discovery benchmarking generally starts with a ground truth mapping of drugs to their associated indications. A variety of data sources are currently in use, including databases, like DrugBank, KEGG BRITE, and the Comparative Toxicogenomics Database (CTD), and pre-extracted mappings, like Cdataset, PREDICT/Fdataset, and the LRSSL dataset. [20, 30, 33, 34, 56-59] Negative drug-indication associations (i.e., non-associations) may be inferred from failed clinical trials, or all associations not in the ground truth may be considered negative, resulting in differing ratios of positive and negative samples. [20] The drug-indication mappings are then usually split into training and testing data. K-fold cross-validation is a comprehensive (every drug-indication association is assessed) and computationally inexpensive (only K rounds of training are required) way of splitting these mappings; it is thus very commonly used. [22-46, 48-53, 60-76] Other protocols, such as a simple training/testing split, a leave-one-out protocol, or a “temporal split” (non-random split based on drugs approved before and after a specific date) are also infrequently used. [76-79] The results of these assessments are then encapsulated in varying metrics. [15] Area under the receiver-operating characteristic curve (AUROC) and area under the precision-recall curve (AUPR) are among the most commonly used metrics as they assess a pipeline at all thresholds. [22-54, 60-73, 75, 76, 78-91] However, the relevance of these metrics to drug discovery remains unclear. [15, 82, 92] More easily interpreted metrics like recall, precision, and accuracy above a certain threshold (e.g., precision at rank 10 or recall with a p-value < 0.05) are also commonly used. [21, 24, 26-28, 33-37, 40, 41, 51, 69, 79, 84, 86, 93-95] Case studies are frequently utilized alongside (and occasionally in the absence of) systematic assessment to provide a more tangible confirmation of predictive power. [22-24, 26-29, 31, 34-54, 61, 65, 66, 68, 69, 73, 76, 79, 82-84, 87, 89, 96-103] The ability of a platform to predict biological properties of small molecules, such as ADMET (absorption, distribution, metabolism, excretion, and toxicity) features, is also assessed on occasion. [87, 95, 97, 103, 104] A lack of benchmarking standards has thus lead to a plethora of data, protocols, and metrics being in use. Our goal for this study is to bring the benchmarking protocols of our drug discovery platform into strong alignment with best practices.

We developed the Computational Analysis of Novel Drug Opportunities (CANDO) platform for multiscale therapeutic discovery. [15, 104-120] CANDO comprises multiple pipelines for drug discovery that vary in the specific protocols and parameters utilized. The fundamental hypothesis underlying CANDO is that drugs with similar multitarget protein interaction profiles or “interaction signatures” will result in similar biological effects. CANDO calculates all-against-all similarities between interaction signatures to predict drug candidates, including repurposing existing drugs for novel uses. [106] Other means of assessing compound similarity, such as chemical fingerprints, may also be used. [118] CANDO and its components have been extensively validated [15, 105, 109-111, 115, 120-130]. Previous efforts to benchmark CANDO have focused on assessing its ability to generate useful drug-drug similarity lists, which are then used to predict novel therapeutic effects for existing drugs. [15, 106, 116, 118] Evaluating the final predictions generated by our platform based on a consensus of these similarity lists should increase the relevance of our benchmarking results to practical application. We therefore updated our internal benchmarking protocol to assess the ability of CANDO to both accurately rank behavioral similarity (as determined by the interaction signature) and incorporate those rankings into effective novel drug predictions. We optimized multiple parameters used in CANDO and examined the influence of certain features on its performance with these revised protocols. Further, we created a head-to-head benchmarking protocol that can be used to consistently assess multiple varieties of drug discovery pipelines, including those within CANDO, an example use of which we present herein. Utilizing the updated protocols and parameters thus created will result in significantly improved performance.

## 3 Methods

### 3.1 Drug discovery using the CANDO platform

The CANDO multiscale drug discovery platform predicts novel compounds for diseases/indications based on the multitarget interaction signatures of the compounds. A signature is an attempt to describe how a compound interacts with biological systems. Every compound is compared to every other compound based on their interaction signatures under the hypothesis that compounds with similar interaction signatures will exhibit similar behaviors. Each compound is thus associated with a sorted “similarity list” that contains every other compound ranked by signature similarity, with lower ranks indicating greater similarity. We used proteomic interaction signatures in this study, which are vectors of predicted compound-protein interaction scores, to evaluate compound-compound signature similarity based on the root mean squared distance between two signatures. [106] CANDO has been described extensively in other publications. [15, 105-107, 112, 114, 116-118, 120]

**Figure 1:**
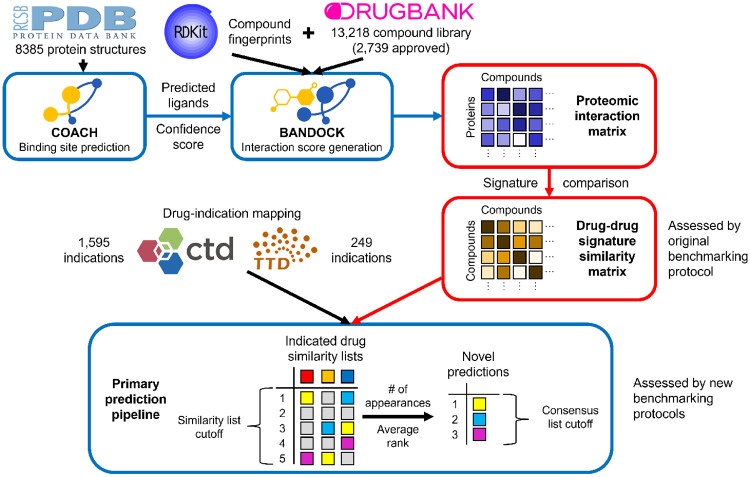
Primary prediction pipeline used in the CANDO platform. The primary prediction pipeline of CANDO is shown, with data sources represented by their respective logos, protocols represented by blue boxes, and key data structures represented by red boxes. COACH is used to predict protein binding sites based off of experimental structures from the Protein Data Bank (PDB) and/or computational models created via tools like I-TASSER. [131] Predicted ligands and confidence scores for each binding site are combined with compound fingerprints (from RDKit) to predict protein interaction scores for every small molecule in the compound library (from DrugBank) using the bioanalytic docking (BANDOCK) protocol. These interaction scores are arranged into interaction signatures for every compound. Drug-drug signature similarity scores are calculated from these signatures. Drug-indication mappings are extracted from the CTD and/or TTD, and the most similar compounds to each drug associated with an indication are examined. Novel compound predictions are generated and ranked based on the number of times a compound appears in these lists above the similarity list cutoff; ties are broken based on average rank in these lists. In the example, the yellow compound is first because it appears the most times, and the cyan compound is second because its average rank is better than that of the magenta compound. The original and new benchmarking protocols differ in what is assessed: the original focuses on the individual similarity lists, whereas the new evaluates the final consensus list.

CANDO uses a consensus protocol to combine multiple similarity lists into novel drug predictions for an indication via the following steps: (1) The similarity lists of any drugs corresponding to the indication (associated drugs) are examined. (2) The most similar compounds to each associated drug are ranked; an adjustable cutoff parameter called the similarity list cutoff determines the number of similar compounds considered for the next step. (3) All compounds are scored based on the number of similar lists in which they appear above the similarity list cutoff (consensus score), with ties broken by their average ranks in those lists. Compounds that are not above the similarity list cutoff (i.e., those with less than the desired similarity to associated drugs) are not considered further. (4) The compounds are sorted by the consensus scores and average ranks. The best ranked compounds in this consensus list are considered to be the top predictions for an indication. The overall prediction pipeline is summarized in Figure 1.

### 3.2 Data extraction and generation

Proteomic interaction signatures were created using predicted compound-protein interaction data. We used the CANDO version 2.5 compound and human protein libraries. The protein library comprised 8,385 nonredundant human protein structures, including 5,316 experimentally determined structures extracted from the Protein Data Bank and 3,069 models generated using I-TASSER version 5.1. [112, 131-134]. Our bioanalytic docking (BANDOCK) protocol requires specific binding site data to calculate compound-protein interaction scores. We used the COACH pipeline to generate these data for our protein library. [135] COACH compared potential binding sites to solved bound protein structures to calculate binding site similarity scores and likely interacting ligands. [110, 135] The chemical similarity between each compound in our library and the most similar predicted ligand of a protein was calculated using ECFP4 chemical fingerprints generated by RDKit. [110, 118, 136] Compound-protein interaction scores were then calculated in three ways: (1) as the chemical similarity score alone (the compound-only or C score), (2) as the product of the chemical similarity score and the binding site similarity score (the compound-and-protein or CxP score), or (3) as the product of the percentile chemical similarity score and the protein binding score (the percentile compound-and-protein or dCxP score). We compared all three interaction scoring types in our protocol optimization study (section 3.4); the second score was used for our predictive power assessment and head-to-head comparison studies.

Benchmarking requires known drug-indication mappings, which we obtained from two sources. We combined drug approval data extracted from DrugBank and drug-indication associations from the CTD to make the “CTD mapping.” [56, 57] These data are also available in version 2.5 of CANDO. The second mapping, the “TTD mapping,” was created from drug approval and indication association data downloaded from the TTD on October 30, 2023. [137] Only approved drug-indication associations were extracted from the TTD, and only drugs already in our compound library were considered. In total, there were: 2,449 approved drugs across 2,257 indications with at least one associated drug and 22,771 associations in the CTD drug-indication mapping; 1,810 drugs across 535 indications and 1,977 associations in the TTD mapping; and 2,739 unique drugs altogether. Of these indications, 1,595 were associated with at least two drugs and thus could be benchmarked in CTD, and 249 were associated with at least two drugs in TTD.

### 3.3 Benchmarking CANDO internally

The original version of the CANDO benchmarking protocol examined the similarity lists of each indication-associated drug [15, 104-120]. Indication accuracy (IA) was calculated as the percentage of similarity lists of associated drugs in which at least one other associated drug appeared above a certain cutoff. Indication accuracies were then averaged for every indication with at least two drugs (required to assess a similarity list) to obtain an overall average indication accuracy (AIA).

We developed a new benchmarking protocol that directly evaluates consensus scoring protocol to more accurately reflect the drug prediction performance of CANDO. This protocol examines each indication with two or more associated drugs. Each associated drug is withheld in turn from its indication and ranked against all compounds to determine whether it would be predicted for that indication if it were not already associated. Next, compounds are ranked by the number of times they appear in the similarity lists of the associated drugs above the similarity list cutoff, resulting in a consensus list. Ties are broken based on the best average rank above that cutoff. Two additional tiebreakers are used to ensure compounds outside of the top ranks are still ordered: (1) best average rank across the similarity lists of the associated drugs and (2) greatest average similarity to the associated drugs.

We determine the rank of each withheld drug in the final, sorted list and calculate multiple metrics to quantify the performance of these consensus lists. New indication accuracy (nIA) is similar to recall, and it is calculated as the percentage of withheld drugs that are predicted as therapeutics for the indication in question at or above the defined rank cutoffs in the consensus list. We set rank cutoffs at 10, 25, and 100 for this study. nIA is then averaged across all indications to calculate the new average indication accuracy (nAIA).

Our protocol also calculates normalized discounted cumulative gain (NDCG), which prioritizes early discovery of true positives and is described in further detail elsewhere. [15] It ranges from zero to one, with a higher score indicating better performance. The discounted cumulative gain can be calculated from the rank of a single associated drug using the following formula:

$$DCG=1∕log_{2}(rank+1)$$

This is divided by the ideal discounted cumulative gain (equal to one in this case) to obtain the NDCG. This metric will be referred to as new NDCG (nNDCG) when calculated by our new benchmarking protocol for the consensus lists. We calculated nNDCG without a rank cutoff (“overall”) and at rank cutoffs of 10, 25, and 100 in this study.

### 3.4 Optimizing a key parameter

We optimized multiple CANDO parameters with regards to the performance of the consensus scoring protocol used for predictions. We randomly split our drug-indication mappings 30/70 to create independent mappings for parameter optimization and performance evaluation, with 30% of drug-indication associations reserved for parameter optimization and 70% for the final assessment. All drug associations with the same indication were assigned to the same group, and only indications with at least two associated drugs were (and could be) assessed. The CTD mapping was split into 5,714 drug-indication associations across 501 indications for parameter optimization and 13,226 associations across 1,094 indications for the final assessment. The smaller TTD mapping was split into 490 associations across 82 indications for parameter optimization and 1,160 associations across 167 indications for the final assessment.

The first parameter optimized was the similarity list cutoff used in our consensus scoring protocol (section 3.1). We quantified the performance using nAIA and nNDCG on the CTD and TTD drug-indication mappings for every value of this parameter up to the number of approved compounds in the mapping (2,449 for CTD, 1,810 for TTD). The similarity list cutoff used when each metric reached its maximum was considered the optimal value. A random control was calculated for each metric and mapping at each optimal value. A hypergeometric distribution was used to calculate the control value for nAIA. For nNDCG, ten randomized drug-protein interaction matrices were generated and benchmarked per optimal parameter and mapping, and the nNDCG values were averaged. We repeated the similarity list cutoff optimization using all 13,218 compounds, approved or otherwise, in the v2.5 CANDO compound library, and similarity list cutoffs up to 1,000 were assessed.

The second parameter optimized was the compound-protein interaction scoring type. We compared all three scoring types used by BANDOCK (section 3.2). We benchmarked CANDO using proteomic interaction matrices generated using each scoring type with similarity cutoffs ranging from 1 to 100, and we compared the best performances of each protocol using nAIA and nNDCG.

The third and final parameter optimized was the tiebreaker used in our consensus scoring protocol. CANDO sorts predicted compounds based on the number of times they appear within the similarity list cutoff in the similarity lists of drugs associated with an indication. Ties are broken by average rank above that cutoff in our original tiebreaker. [106] In benchmarking, we also use the overall average rank, the average rank of a compound in the full similarity lists (i.e., not limited to the similarity list cutoff), as a secondary tiebreaker to ensure that all compounds are ranked. The summed similarity score is used as a final tiebreaker. We compared average rank within the similarity list cutoff to overall average rank by benchmarking CANDO with similarity list cutoffs ranging from 1 to 100 using overall average rank as the primary tiebreaker and average rank within the cutoff as the secondary tiebreaker. Performance was evaluated using nAIA and nNDCG.

### 3.5 Evaluating features affecting performance

A final assessment was completed using the 70% of indications not used for parameter optimization. Similarity list cutoffs, interaction scoring types, and tiebreakers were chosen based on parameter optimization results (section 3.4): similarity list cutoffs of six, ten, and thirteen, the compound-and-protein score, and average rank above the similarity list cutoff were used. We calculated nAIA and nNDCG at rank cutoffs of 10, 25, and 100, in addition to overall nNDCG, in this final assessment.

We examined how multiple features correlated with performance, including our previous benchmarking metric (AIA), the number of drugs associated with an indication, and the chemical similarity of the drugs associated with an indication. The correlation between these features and performance were considered at the drug scale using the rank at which each individual drug was predicted and at the indication scale using nIA and nNDCG. Rankings are ordinal, and our metrics are unlikely to follow a normal distribution, which violates the assumptions of Pearson correlation. Therefore, Spearman correlation coefficients were calculated using the scipy package. [138] For brevity, correlation results are reported for a similarity list cutoff of ten only.

AIA, which measures similarity list quality, was calculated using our original benchmarking protocol (section 3.3). [106] The correlation between the rank of a drug associated with an indication using our new benchmarking protocol (i.e, the rank in the consensus list) and the best rank of another associated drug in its similarity list was calculated. The correlations between IA and nIA at the top10, 25, and 100 cutoffs were also calculated. Correlation coefficients were re-calculated when only considering indications with a certain number of associated drugs: those with two associated drugs (208 indications in CTD, 71 in TTD), with four or fewer drugs (485 in CTD, 109 in TTD), and with five or more drugs (609 in CTD, 58 in TTD).

We examined the relationship between the number of drugs associated with an indication (indication size) and performance using nIA and nNDCG. Including associated drugs in the consensus list would negatively bias performance for indications with more associated drugs. For example, for an indication with 101 associated drugs, a withheld drug would need to outcompete every other associated drug, all of which should be ranked highly for that indication, to be ranked in the top 100. However, excluding associated drugs positively biases performance for larger indications; in the previous example, the withheld drug would need to outcompete 99 fewer drugs than if it were in an indication with 2 associated drugs. We therefore calculated nIA and nNDCG including all associated drugs in the consensus list, and we also re-calculated these metrics while excluding all associated drugs but the withheld drug from the rankings. The unbiased value should fall between these two measurements. In both cases, we measured the overall correlation and the correlation for only indications with five or more drugs.

Lastly, we examined the influence of drug chemical similarity within an indication on the performance of CANDO, expanding on previous work. [118] Drug-drug chemical signature similarity was measured using the Tanimoto coefficient using 2048-bit Extended Connectivity Fingerprints with a diameter of 4 (ECFP4) vectors that encode the chemical features of a compound, which were generated by RDkit to represent each drug.[136, 139] The best and average similarities of each individual drug to every other drug associated with the same indication were calculated. The correlation between these metrics and the rank of that drug in the consensus list generated by our benchmarking protocol was determined. Three similarity metrics were also calculated for each indication: best similarity between any pair of associated drugs, average of the best similarities of the associated drugs, and average of the average similarities of the associated drugs. We calculated the correlation between these per-indication metrics and nIA and nNDCG, respectively. Finally, we benchmarked the performance of CANDO using the ECFP4 chemical signature similarity in place of interaction signature similarity.

### 3.6 Comparing drug-indication mappings

We examined the effects of the drug-indication mapping used on performance by comparing the mappings extracted from CTD and TTD. We combined the drugs from both mappings into a single drug library and re-benchmarked CANDO on this library using each mapping. We manually matched each TTD indication to the most appropriate CTD indication for comparison purposes. When no appropriate CTD indication match existed, for instance, for the TTD indication “Contraception,” that indication was excluded from the comparison. When multiple TTD indications were initially mapped to the same CTD indication, only the most similar TTD indication was matched: for instance, “Open-angle glaucoma” in TTD was matched to “Glaucoma, Open-Angle” in CTD, so “Chronic open-angle glaucoma” was not. The difference in performance using nIA and nNDCG between the two mappings was evaluated for the matched indications. Average performance on the matched and unmatched indications was also calculated. Finally, we compared the rankings of the drugs that appeared in the same indications in both CTD and TTD.

### 3.7 Benchmarking platforms head-to-head

The CANDO platform consists of both similarity-based and non-similarity-based pipelines for novel drug prediction. [106] We focused on the similarity-based pipelines in this study, which have specific benchmarking requirements. However, other platforms or pipelines may have other requirements; thus, we created compbench, a protocol for head-to-head benchmarking of drug discovery platforms in general. This protocol will ease comparison of disparate pipelines and platforms, including those within CANDO and those created by others. Our head-to-head benchmarking protocol uses k-fold cross-validation to accommodate pipelines that are computationally expensive or slow to train. Drug-indication associations are randomly split into a number (k) of equally sized subsets (folds), one of which is used for testing and the remainder of which are used for training. Assessment is repeated once per fold and the results from all fold assessments are averaged. We stratified this splitting by indication: drugs from each indication are randomly, but evenly, distributed between folds. This ensures that there is consistent training data available for each indication in each fold. Indications with fewer than two associated drugs are excluded from assessment, as before. The number of folds used can be set as desired; for this study, we used ten.

We used metrics that are widely applicable, comparable, and useful for our head-to-head comparison. Area under the receiver operating characteristic curve (AUROC) is commonly used for holistically assessing computational models. [15] However, only the most selective thresholds are practically useful for drug discovery. This has led some to suggest calculating AUROC up to a maximum false positive rate cutoff. [92] Therefore, we assessed on both traditional AUROC and partial AUROC up to a false positive rate of 0.05. We also consider NDCG useful as it prioritizes early retrieval of effective therapies, and it is applicable to any platform that generates ranked predictions. We considered NDCG without a cutoff and with a rank cutoff of ten as our final two metrics for this assessment. These metrics were calculated based on the ranks at which the withheld drugs were recovered for their corresponding indications. We used a theoretical random control with a slope of 0.5 for AUROC, and we scored the ranks created by randomly shuffling our compound list to create a random control for nNDCG.

Compbench is publicly available as a Python script. The protocol gives a set of indication-associated drugs and a set of other compounds, including any withheld drugs, as input to the drug discovery pipeline or a wrapper thereof; other input may be provided as necessary. It must then receive the list of other compounds sorted by likelihood of efficacy for the indication (greatest to least). Data splitting and metric calculation is automatically completed as outlined above. The code is available on Github at https://github.com/ram-compbio/compbench and on our server at http://compbio.buffalo.edu/software/compbench/; the cross-validation data used for this assessment is available in both places as well.

#### 3.7.1 Assessing the subsignature pipeline

To fully demonstrate the above head-to-head benchmarking protocol, we created a pipeline that was sufficiently dissimilar to our primary one. We chose a pipeline that predicts novel drugs based on subsignature similarity. This involves splitting the complete proteomic signature into shorter subsignatures based on the Gene Ontology terms mapped to each protein. [140, 141] Gene Ontology-protein associations were extracted from UniProt. [142] A protein associated with a term was also considered to be associated with its parent terms in the Gene Ontology hierarchy. We used 650 higher-level Gene Ontology terms that mapped to at least one protein as the basis for our subsignatures.

The compound ranking protocol of the subsignature pipeline involves the following steps: (1) The subsignatures of the drugs associated with an indication are clustered. The number of clusters is chosen based on repeated assessment of cluster centrality using an adapted version of the kneedle knee/elbow-finding protocol. [143] This protocol uses the curvature of a cost/benefit graph to find the point at which increased cost (additional clusters) is no longer worth the benefit (increased centrality). The cluster number is limited to 20% of the compounds associated with an indication or ten, whichever was lower. (2) The similarity between the compound subsignatures and the indication clusters corresponding to the same Gene Ontology terms are calculated. (3) These similarities are summed in one of three ways: unweighted, weighted by the negative logarithm of cluster centrality (log weighted), or weighted to only consider the 25 most central clusters (25 weighted). (4) The compounds are ranked by this summed similarity from most to least similar.

The subsignature pipeline was benchmarked on the CTD and TTD drug-indication mappings using compbench. The code for this version of the subsignature pipeline, the wrapper used to make it compatible with compbench, and the associated data (including Gene Ontology terms used) can be accessed at http://compbio.buffalo.edu/software/compbench.

#### 3.7.2 Assessing the primary pipeline

We also benchmarked the primary drug discovery pipeline of CANDO using compbench. We used overall average rank and summed similarity as additional tiebreakers in our internal benchmarking protocol. This ensured that the consensus list as a whole was sorted, rather than only those compounds that appeared above the similarity list cutoff being sorted. However, doing this required changing the internal protocols of CANDO; we cannot do this in our head-to-head protocol as it is not part of CANDO. Instead, we needed to use features and parameters already present in CANDO to create a full ranked list. Therefore, we created three different pipeline variants based on varying the similarity list cutoff to create a full ranked list: First, we used a similarity list cutoff equal to the total number of compounds (all similar variant), resulting in compounds being sorted based on their overall average rank as they all have the maximum possible consensus score. Second, we used a similarity list cutoff of ten (ten similar variant), with any compounds not appearing in the top ten compounds being sorted by average overall rank. Finally, we created predictions using multiple similarity list cutoffs {10, 20, 30…} (multiple lists variant), combining the lists so that compounds recovered at lower similarity list cutoffs had better ranks than those recovered at higher cutoffs.

All three variants were assessed on the CTD and TTD drug-indication mappings using compbench. The wrappers used to integrate CANDO with this benchmarking protocol and the data used in this assessment can be accessed at http://compbio.buffalo.edu/software/compbench/.

## 4 Results and discussion

In this study, we created two new benchmarking protocols to allow more consistent assessment of CANDO and computational drug discovery platforms in general. We present results obtained via these new protocols, including (1) the optimization of multiple key parameters involved in our drug prediction protocol; (2) an assessment of the performance of CANDO using these optimized parameters, including the correlations between performance on the new benchmarking protocol and the number of drugs associated with a disease/indication, the results of our original benchmarking protocol, and the drug-drug chemical signature similarity within an indication; (3) a comparison of performance when using two different drug-indication mappings as a ground truth; and (4) the application of compbench, a novel tool for generalized and head-to-head benchmarking of drug discovery platforms, to a comparison of the primary pipeline of CANDO and a novel pipeline in development, the subsignature pipeline.

### 4.1 Optimization of three key CANDO parameters

Our new internal benchmarking protocol allows us to directly assess the performance of the consensus scoring protocol used in CANDO to rank potential therapeutics (section 3.3). This allowed us to improve CANDO by optimizing a key consensus scoring parameter, assessing the effects of the protein interaction scoring protocol used, and comparing two different ways of breaking ties when ranking novel compound predictions.

CANDO requires a similarity list cutoff when generating predictions; this determines how many similar compounds the consensus scoring protocol considers per drug associated with the indication when predicting new drugs or benchmarking. This parameter was set to ten by default, but various values have been used in previous applications of CANDO. [104, 109, 111, 115, 119, 120]

**Figure 2:**
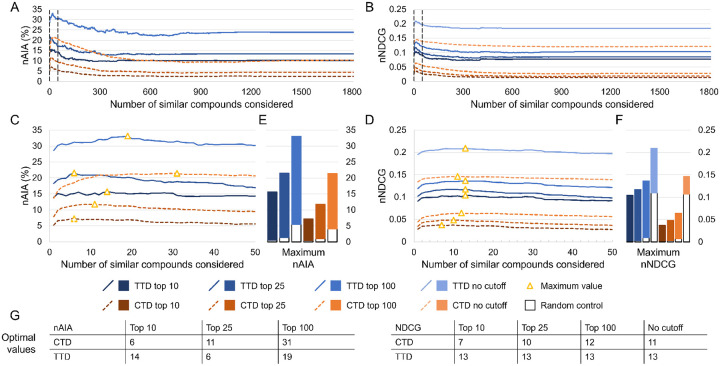
Effects of the similarity list cutoff on benchmarking performance. We used our new benchmarking protocol to optimize the similarity list cutoff parameter, which represents the number of similar compounds the consensus protocol considers per associated drug when predicting a new compound for an indication. Assessments were completed on two drug-indication mappings extracted from the CTD and TTD. Results were summarized using nAIA and nNDCG metrics at multiple rank cutoffs; nNDCG was also calculated without a rank cutoff. Performance using nAIA (A) and nNDCG (B) is shown for similarity list cutoffs up to 1,810. Dotted black lines indicate the cutoffs of 1 and 50, between which all optimal values for this parameter fall. An expanded graph of only this range is shown for nAIA (C) and nNDCG (D). Bar charts (E–F) show the maximum values of each metric against random controls. Optimal values are marked with a yellow triangle and listed in the tables (G) at the bottom. The optimal parameter values for nAIA varied from 6 to 31 based on the cutoff and mapping used. The range was smaller for nNDCG, ranging from 7 to 13. The similarity list cutoff affected performance on multiple key metrics, and optimal performance was only achieved when less than 2% of compounds were considered.

We also compared the performance of CANDO on subsets of drug-indication mappings extracted from the CTD and TTD. Results were quantified using new average indication accuracy (nAIA) and new normalized discounted cumulative gain (nNDCG) metrics at top10, top25, and top100 rank cutoffs; nNDCG was also calculated without a rank cutoff. The results for similarity list cutoffs up to 1,810 are shown in Figure 2.

The performance of CANDO varied widely based on the similarity list cutoff used. The largest gap between the best and worst performances was observed in the CTD mapping for nAIA top100, with the best nAIA being 21.4% (similarity list cutoff of 31) and the worst nAIA being 9.2% (cutoff of 805). CANDO outperformed the random control in all cases, including when there was suboptimal performance. Different optimal parameter values were obtained for different metrics and drug-indication mappings, ranging from 6 (nAIA top10 using CTD and nAIA top25 using TTD) to 31 (nAIA top100 using CTD). Performance was better with both the nAIA and nNDCG metrics and at all cutoffs when using the TTD mapping relative to the CTD mapping. The optimal similarity list cutoff for both mappings was greatest when considering nAIA top100, and the range of optimal values was greater for nAIA (6 to 31) than for nNDCG (7 to 13). For instance, the optimal similarity list cutoff was 13 for all four nNDCG cutoffs when using the TTD mapping. This may be because the inherent prioritization of top-ranked hits in the calculation of NDCG makes the consensus list cutoff used matter less.

In general, we only rank approved compounds during benchmarking so that the results are not be dependent on the number of unapproved compounds included. However, there are numerous unapproved small molecules that could potentially have novel therapeutic uses, and it is often desirable to use CANDO with a compound library that includes such small molecules in hopes of finding a completely novel therapeutic. Therefore, we repeated this optimization assessment on the full 13,218 compound library in CANDO version 2.5, which includes experimental/investigational drugs, to obtain a parameter value relevant to this scenario (Supplementary Table 1A). Performance overall decreased with the inclusion of additional compounds without any associated indications, and the optimal parameter values were also affected. The optimal parameter value increased for twelve of the fourteen metric/mapping combinations used, with the largest increase being 27 (from 6 to 33, nAIA top10 using CTD). This demonstrates the necessity of considering application conditions when completing benchmarking assessments and making corresponding predictions.

Another feature that we assessed is the protocol used to calculate the drug-protein interaction scores required to generate drug-proteome interaction signatures. These signatures are compared to produce drug-drug interaction signature similarity scores. Our BANDOCK interaction scoring protocol (section 3.2) computes three types of interaction scores: compound-only, compound-and-protein, and percentile compound-and-protein. We optimized the similarity list cutoff using nAIA and nNDCG with all three interaction scoring types and compared the best values for each benchmarking metric (Supplementary Table 1B). The compound-and-protein type showed the best performance on most benchmarking metrics when using the drug-indication mapping from CTD, with the percentile compound-and-protein protocol performing the best on the remaining metrics (nNDCG top10, overall nNDCG). On the other hand, the compound-only protocol performed best on the majority of metrics when using the TTD mapping, with the compound-and-protein protocol performing best on one (nAIA top25). The compound-and-protein scoring type was often the best performing one, and never the worst performing; we therefore consider it the optimal type of protein interaction score for use with CANDO.

Finally, we examined the impact of the tiebreakers used in our consensus scoring protocol (section 3.1). Following sorting by the consensus score (section 3.3), our original tiebreaker took the average rank of a drug within each similarity list limited to the similarity list cutoff. We compared this to using overall average similarity rank without using the cutoff (Supplementary Table 1C). Average rank within the cutoff performed better than the overall average rank for all metrics in both mappings. nAIA was 2.9% (nAIA top100 using TTD) to 11.8% (nAIA top10 using CTD) higher and nNDCG was 2.4% (nNDCG overall using TTD) to 12.3% (nNDCG top100 using CTD) higher when average rank within the cutoff was used as the primary tiebreaker.

### 4.2 Assessment of predictive power

Our new benchmarking protocol also allowed us to obtain a more accurate estimation of the predictive power of CANDO. We used parameters based on our optimization results to conduct three assessments with similarity list cutoffs of six, ten, and thirteen to cover the variety of optimal values obtained. We used the compound-and-protein interaction scoring type as it was often the best performing one and never the worst performing. Finally, we used average rank within the cutoff in the consensus scoring protocol as it was dominant in our optimization assessment (section 4.1).

**Figure 3:**
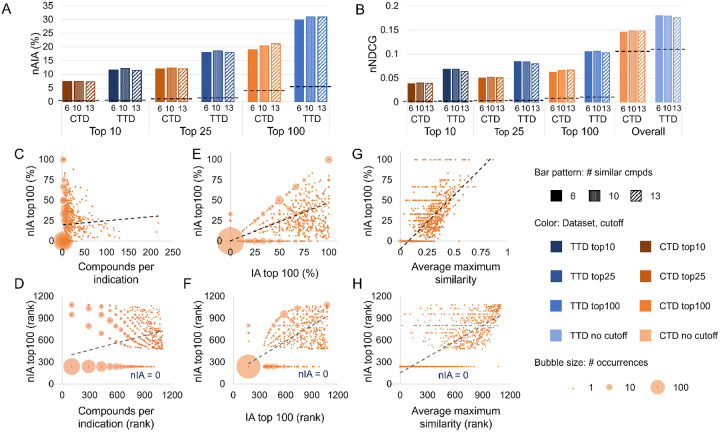
Assessment of predictive power. CANDO was assessed using the protocols and parameters obtained through our optimization. nAIA (A) and nNDCG (B) metrics are shown at multiple cutoffs for the two drug-indication mappings, CTD and TTD. The random control is shown as a dotted line on each group of bars with the same mapping and cutoff. CANDO outperformed the control on all assessments, and performance was best when using the TTD mapping. Performance on this assessment was correlated with multiple features: the number of compounds in an indication (C–D); our original indication accuracy (IA) metric, which measures similarity list quality (E–F); and drug-drug interaction signature similarity within each indication, measured as the average similarity score between each drug and its most similar other associated drug (G–H). The upper subfigures (C, E, and G) plot each feature against new indication accuracy (nIA) above rank 100 in CTD, whereas the lower plots (D, F, and H) show the relationship between the same two features when their values are ranked; these ranks were used to calculate Spearman correlation coefficients. The size of the bubble surrounding each dot represents the number of indications plotted there. Trendlines are shown as dotted black lines. Positive correlations of varying strength were observed in all cases. Knowledge of the features influencing benchmarking can enable more accurate assessment of expected predictive performance. Based on these results, we can expect CANDO to perform best when predicting compounds for indications with large numbers of associated drugs and when chemical signature similarities are relatively high.

We assessed the overall performance of CANDO using drug-indication associations that were not used for optimization. The results are shown in Figure 3A-B. CANDO outperformed random controls when using both drug-indication mappings and for all metrics. The nAIA results suggest that CANDO recovered approximately 7.3% to 7.4% of approved drugs within the top 10 compounds when using the CTD mapping and 11.4% to 12.1% when using the TTD mapping (out of 2,449 in CTD and 1,810 in TTD). This rose to 19.0% to 21.1% when using CTD and 29.9% to 31.0% when using TTD at the top 100 cutoff. nNDCG top10 ranged from 0.038 to 0.040 using CTD and 0.061 to 0.066 using TTD, more than an order of magnitude greater than the corresponding random control values. Complete performance data for all similarity list cutoffs are available in Supplementary Table 2.

Performance increased by up to 11.2% (nAIA top100 using the CTD mapping) and at least 1.9% (overall nNDCG using CTD) between the worst and best performing similarity list cutoff used. Performance also differed from what we observed during parameter optimization. Performance on this assessment using CTD was 1.2% (NDCG overall, similarity list cutoff of 6) to 7.8% (NDCG top10, cutoff of 10) better than performance at the same similarity list cutoff on the optimization assessment. The change was more extreme and more negative using the TTD mapping: performance decreased by 0.4% (nAIA top100, cutoff of 10) to 38.8% (nNDCG top10, cutoff of 13) when using the TTD mapping. The increase using CTD and decrease using TTD made the difference in performance between the mappings more similar, but performance was still consistently and substantially higher when using the TTD mapping. In five out of seven assessments using CTD (nAIA and nNDCG at the specified cutoffs), the similarity list cutoff that was closest to the previously observed optimal value showed the best performance. However, the previous optimal value and best performance on this assessment did not align for any assessment on TTD. This and the up to 38.8% decrease in performance on this assessment suggest that our random splitting of the TTD mapping resulted in somewhat dissimilar indication libraries for optimization and assessment. The smaller size of the TTD mapping may have also contributed to this difference, demonstrating the need for large and robust benchmarking ground truth datasets for drug discovery.

#### 4.2.1 Influence of the number of associated drugs

We investigated three features that could influence the performance of CANDO to understand what makes it perform better on some indications than others. First, we considered the influence of the number of approved drugs associated with an indication (or “indication size”) on performance. We include all associated drugs in the performance assessments of our new benchmarking protocol by default. However, performance may be negatively impacted when there are more drugs associated with an indication since other associated drugs will be competing with the one being withheld and assessed (section 3.5), biasing the correlation coefficient. On the other hand, excluding the non-withheld drugs associated with the indication would positively bias results as there would be fewer total compounds being ranked against the withheld drug. Therefore, we assessed CANDO once when including other associated drugs and once when excluding them, and we calculated two correlation coefficients per assessment. The actual correlation should fall between the positively biased and negatively biased coefficients so measured.

Greater data availability generally improves the performance of computational models, so we anticipated a positive correlation between nIA and indication size. Indeed, there was a weak positive correlation, with Spearman correlation coefficients ranging from 0.324 to 0.352 using the CTD mapping and from 0.337 to 0.505 using the TTD mapping when associated drugs were included in rankings. Coefficients raised only slightly when associated drugs were excluded, ranging from 0.326 to 0.355 using the CTD mapping and 0.342 to 0.511 using the TTD mapping. The correlation between nIA at the top100 cutoff and indication size when using the CTD mapping and excluding associated drugs is illustrated in Figure 3C-D. Correlations using nIA at the top10, 25, and 100 cutoffs using both mappings are shown in Supplementary Figure 1.

Although there was a positive Spearman correlation, from visual inspection of the datapoints in Figure 3C-D alone, one might anticipate a neutral or negative correlation between indication size and performance using CTD. We observed a large number of indications with few approved drugs that had an nIA of zero, so we hypothesized that the positive correlation may be largely due to low performance on indications with very few associated drugs. We thus re-calculated the correlation coefficient using only indications with five or more associated drugs (Supplementary Table 3B). The strength of the correlation between nIA and indication size weakened in this assessment, becoming negligible at 0.026 to 0.075 when using the CTD mapping with associated drugs excluded. Correlation coefficients also shrunk when using the TTD mapping, ranging from 0.161 to 0.281. This suggests that, particularly when using the CTD mapping, increasing indication size may not improve performance beyond a certain point, for example, when nonzero performance has been achieved.

This could be because indications with more associated drugs may include those with disparate mechanisms of action. This would lower drug-drug interaction signature similarity within the indication, decreasing performance. Relatedly, indications with more associated drugs could contain multiple related, smaller indications, which would lower drug-drug interaction signature similarity between drugs aimed at these different sub-categories. For example, drugs intended to treat “Breast cancer” may actually be aimed at treating HER2-positive breast cancer, triple negative breast cancer, metastatic breast cancer, and so on. Finally, it could be because larger indications may be more likely to include at least one spurious drug association. This would also explain why the correlation between indication size and performance was weaker when using the CTD mapping, which includes drug-indication assocations drawn from the literature, than when using the TTD mapping, in which associations were based on the stricter standard of FDA approval.

#### 4.2.2 Influence of similarity list quality

We also considered the primary metric of our previous internal benchmarking protocol: indication accuracy (IA; section 3.3). IA directly measures the quality of the drug-drug interaction signature similarity ranks calculated within CANDO. Note that IA is more lenient than nIA as it checks whether at least one other associated drug appears above a certain cutoff in the similarity list of a drug rather than the percentage of associated drugs recalled. IA thus tends to have higher values than nIA when assessing the same indication.

We calculated the Spearman correlation coefficient between the rank assigned to each drug in the consensus list and the best rank of a drug associated with the same indication in its similarity list, as calculated by the original benchmarking protocol. There was a moderate-to-strong correlation between the two ranks, with a correlation coefficient of 0.592 when using the CTD drug-indication mapping and 0.704 when using the TTD mapping. We also examined the relationship between nIA and IA at the top10, 25, and 100 cutoffs for each indication. This correlation was even stronger, with coefficients ranging from 0.741 to 0.807 using the CTD mapping and 0.859 to 0.905 using the TTD mapping. There was no consistent relationship between the cutoff considered and the strength of the correlation. The correlation between nIA and IA at the top100 cutoff using the drug-indication mapping from CTD is illustrated in Figure 3C-D. The remaining correlations are shown in Supplementary Figure 2. The correspondence between IA and nIA suggests that our previous benchmarking results did have relevance to actual performance, as has also been demonstrated by extensive prospective validation. [15, 105, 109-111, 115, 120-130] This also, unsurprisingly, suggests that high-quality similarity lists result in high-quality consensus predictions.

A stronger correlation between IA and nIA was observed using the TTD drug-indication mapping. We hypothesized that this may have resulted from a difference in the number of drugs associated with each indication on average in the two mappings: indications in the CTD mapping were associated with 12.1 drugs on average compared to 6.9 drugs in the TTD mapping. We calculated the correlation coefficient between IA and nIA when only considering indications associated with up to four drugs and with at least five drugs to test this (Supplementary Table 3A). Correlation coefficients were higher when only indications with up to four associated drugs were included (485 indications in CTD and 109 in TTD), ranging from 0.831 to 0.925 using CTD and from 0.879 to 0.948 using TTD. Meanwhile, correlation coefficients were lower when only indications associated with five or more drugs were considered (609 indications in CTD and 58 in TTD), ranging from 0.476 to 0.635 using CTD and from 0.419 to 0.636 using TTD. These results can be explained by the increased impact of each individual similarity list (assessed using IA) and decreased impact of the consensus scoring protocol (assessed using nIA) in indications with fewer associated drugs. This indicates that benchmarking the consensus scoring protocol directly, as done in this study, is important to accurately capture performance on indications with a greater number of associated drugs.

#### 4.2.3 Influence of chemical signature similarity

CANDO typically uses the proteomic interaction signature similarity between indication-associated drugs and other compounds to predict whether those compounds would be effective for that indication. Compounds that are alike in chemical structure tend to have similar protein interactions and, therefore, similar interaction signatures. [118]

We assessed the extent to which chemical similarity, calculated as the Tanimoto coefficient between the chemical fingerprints of two drugs, influences the performance of CANDO. [139] There were moderate correlations of −0.589 when using the CTD mapping and −0.618 when using the TTD mapping between the rank at which a drug was recalled and its average chemical similarity to other drugs associated with the same indication. The negative coefficients indicate that lower (better) ranks correspond with higher similarity. The strength of the correlations were similar or slightly stronger when using maximum similarity to any other associated drug: −0.593 using the CTD and −0.671 using the TTD mapping.

Next, we examined the correlation between indication-wide similarity and performance using nIA and nNDCG. We quantified indication similarity through three metrics: maximum chemical similarity between any pair of associated drugs; average chemical similarity across all pairs of associated drugs; and the average of the maximum chemical similarities of each associated drug. The correlation was strongest using average maximum similarity, for which coefficients ranged from 0.635 to 0.750 using CTD and 0.697 to 0.744 using TTD (Supplementary Table 3C). The greater correlation between performance and average maximum similarity as compared to overall average similarity suggests that CANDO does not require an indication be totally chemically homogeneous to perform well, but that it performs best when drugs have at least one chemically similar partner in the same indication. The correlation between nIA top100 and average maximum similarity using the CTD mapping is illustrated in Figure 3G-H, and correlation with nIA top10, top25, and top100 using both mappings are shown in Supplementary Figure 3.

We examined whether using proteomic interaction signature similarity confers an advantage over chemical signature similarity since the latter had a moderate-to-strong relationship with the performance of CANDO, even when using the proteomic signature. We reassessed CANDO using chemical signatures in place of proteomic interaction signatures. [118, 139] Performance was slightly worse when using the chemical signature; for example, nIA top10 decreased from 7.4% to 5.9% using the CTD mapping and from 12.1% to 10.9% using the TTD mapping. This represents a 20.5% decrease when using CTD and a 9.8% decrease when using TTD, respectively. A similar decrease was observed at the other nIA cutoffs and for nNDCG; the exception were nNDCG overall using both mappings and nNDCG top10 when using TTD mapping only. This result demonstrates that, though the protein interaction scores of compounds are calculated based on their chemical signatures and though compound chemical similarity correlates with performance, the use of the protein interaction signature adds value to the performance of CANDO.

### 4.3 Comparison of drug-indication mappings used

CANDO consistently performed better in the above assessments when using the drug-indication mapping created from the TTD relative to the one from the CTD. These databases differ from one another in a few ways. Drug-indication associations in the CTD are curated from evidence of a therapeutic association in the literature. The associations in TTD are based on FDA approval instead, which is a higher standard of evidence. This may lead to higher quality associations, which could improve the performance of CANDO. CTD contains indications with more associated drugs on average, which should result in improved performance based on the observed positive correlation with indication size. It also includes more total approved drugs, which means that the assessed drug has to outcompete more compounds during benchmarking and leads to decreased performance (as observed in the lower random control when using the CTD mapping). CTD and TTD also contain different indications. If the indications in the TTD mapping are easier to predict drugs for on average, this could also explain the better benchmarking performance when using the TTD mapping.

**Figure 4:**
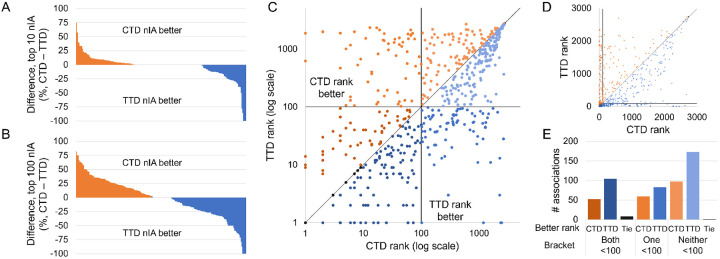
Influence of drug-indication mapping on performance. CANDO was benchmarked using drug-indication mappings extracted from two databases, CTD and TTD. The differences in nIA at the top10 (A) and top100 (B) cutoffs for each indication that appeared in both mappings are shown. Performance was better using CTD for more indications, but TTD outperformed by more when it was superior. This lead to a higher overall nAIA when using TTD. We also compared the ranks of 576 drug-indication associations that appeared in both mappings. The ranks of those drugs when predicted or their indications in each mapping are plotted in log scale (C) and arithmetic scale (D). Black lines indicate the 100^*th*^ rank, beyond which predictions are less likely to be useful for drug discovery, and a grey line represents equivalent ranks between the mappings. The number of associations for which each mapping performed better is shown (E); these counts are separated by whether both, only one, or neither mappings ranked the drug within the top100 cutoff. TTD outperformed CTD on more drugs than vice versa. A drug was more likely to rank well for its indication when using the TTD drug-indication mapping, but more individual indications performed better at the most stringent top10 cutoff when using the CTD mapping.

We examined the two drug-indication mappings head-to-head to determine whether using the TTD mapping with CANDO actually results in improved performance. We benchmarked CANDO on both drug-indication mappings using the full library of drugs that were marked as approved in either mapping. CANDO still performed better when using the TTD mapping, with a top10 nAIA of 6.8% using CTD compared to 11.3% using TTD and a top100 nAIA of 19.1% using CTD compared to 27.5% using TTD. However, this difference decreased when we only considered the 191 indications that appeared in both mappings. The top10 nAIA using CTD with matched indications was 6.5% compared to 9.3% using TTD, and the top100 nAIA using CTD was 24.5% compared to 26.4% using TTD. The differences in performance per matched indication using nIA top10 and top100 are shown in Figure 4A-B. The CTD mapping performed best on more indications than the TTD mapping, but the TTD mapping generally outperformed by a greater magnitude, leading to its higher nAIA.

The top10 nAIA was 21.5% greater when the full TTD mapping was used compared to assessing on only TTD indications also present in the CTD mapping. Likewise, the top10 nAIA was 17.6% when only the 51 indications *not* matched to CTD were considered, an increase of 55.8% over that observed when using the full TTD mapping. CTD had only a slight performance gap: its top10 nAIA was 6.5% on matched indications and 6.8% on both unmatched indications and the full CTD mapping. TTD indications that had high nIAs and did not appear in the CTD mapping included anesthesia (ICD-11 9a78.6; 33 drugs, 15.1% top10 nIA) and contraception (ICD-11 qa21; 10 drugs, 30% top10 nIA). Virus infection (ICD-11 1a24-1d9z; 8 drugs, 50% top10 nIA) appeared in both mappings, but it was only associated with one drug in the CTD mapping and was thus only benchmarked using the TTD mapping. The higher performance of TTD on indications not in CTD suggests that the apparent better performance when using TTD is, in part, due to its inclusion of “easier” indications, ones that CANDO more accurately predicts novel drugs for. However, this cannot be the only factor as performance using the TTD mapping was still higher when only indications in both mappings were considered.

We then examined drugs that were associated with the same indications in both mappings. There were 576 drug-indication associations that appeared in both CTD and TTD; the rankings assigned to these drugs by our benchmarking protocol when using each mapping are plotted in Figure 4C-E. Of these drugs, 208 had better ranks when using CTD, 359 were better when using TTD, and 9 had the same ranks in both scenarios. However, CTD generally outperformed TTD by a greater magnitude when it was the better performer, as can be seen in the distance of the dots from the center line in Figure 4C. Still, the average difference in rank between the mappings was 23.5 in favor of TTD. Drugs associated with the same indications in both the CTD and TTD mappings were more likely to be ranked in the top10, 25, and 100 cutoffs when using TTD.

Overall, these results support the hypothesis that using the current TTD mapping improves performance, likely due to the higher standard of evidence for inclusion in this mapping (section 3.2). Benchmarking and prediction generation using the TTD mapping may thus provide more meaningful and reliable results for certain indications. That being said, the CTD mapping contains more total indications than the TTD mapping, and many individual indications showed better performances when using this mapping. The CTD mapping is still useful for such indications not present in the TTD mapping or with poor benchmarking performance using TTD. This demonstrates another justification for the development of rigorous benchmarking protocols: benchmarking allows us to create optimal parameter and mapping combinations for our predictions on a case-by-case or indication-by-indication basis.

### 4.4 Case study of head-to-head benchmarking

We designed compbench, a head-to-head benchmarking protocol, to facilitate consistent comparison of different drug discovery platforms, including CANDO. We compared the performance of the primary pipeline of CANDO with a new pipeline we are developing (the “subsignature pipeline”) as a case study of compbench; three variants of each pipeline were assessed to give both pipelines multiple opportunities to perform at their best (section 3.7).

The primary pipeline in CANDO outputs a ranked list of compounds that appear above a chosen similarity list cutoff at least once by default, which results in not all compounds being ranked. We therefore utilized the similarity list cutoff in three ways to create the all similar, ten similar, and multiple lists variants (section 3.7.2). We also created three variants of the subsignature pipeline that use different scoring types: the unweighted, log weighted, and 25 weighted variants (section 3.7.1).

**Figure 5:**
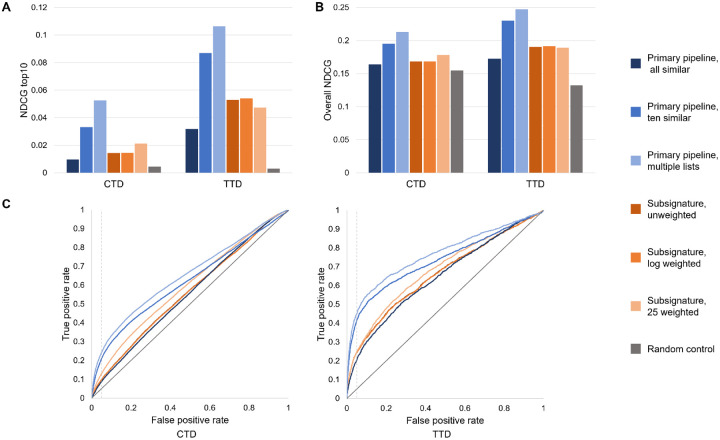
Head-to-head comparison of disparate drug discovery technologies. Three variants each of our primary drug discovery pipeline and the subsignature pipeline, which uses smaller interaction signatures of proteins grouped by common Gene Ontology terms, were benchmarked. All six were assessed using both the CTD and TTD drug-indication mappings. Top10 (A) and overall (B) NDCG were calculated, and the receiver operating characteristic curves (ROC) were plotted (C). A random control NDCG is shown for each mapping, and the theoretical random ROC is plotted. A vertical line in (C) marks a false positive rate of 0.05, which was used to calculate partial area under the ROC (AUROC); overall AUROC was also calculated. Performance was better for all pipelines when using TTD relative to CTD. The primary pipeline outperformed the subsignature pipeline with the exception of the “all similar” variant, which uses a suboptimal similarity list cutoff. All pipelines and variants outperformed random control. Though the primary pipeline was superior when used optimally, the subsignature pipeline still outperformed its least optimized variant, suggesting that this relatively new pipeline may be able to catch up with further optimization.

We assessed all six variants using the CTD and TTD mappings (section 3.7). We quantified the performance of each on top10 NDCG, overall NDCG, AUROC above a false positive rate of 0.05 (“partial AUROC”), and overall AUROC. The results of these assessments are shown in Figure 5. All pipeline variants performed better when using the TTD mapping relative to using the CTD, which is consistent with our internal benchmarking results. The remainder of this section will therefore focus on the TTD results, as those results show each variant at its best. Similar patterns were observed in the CTD results.

All variants performed above random chance on all metrics examined and using both drug-indication mappings. The multiple lists variant, which combines the output of the primary pipeline when run with multiple similarity list cutoffs (section 3.7.2), had the best performance, with a top10 NDCG of 0.106, overall NDCG of 0.247, partial AUROC of 0.0163, and overall AUROC of 0.767. There was no single best-performing subsignature variant, with the unweighted and log weighted variants slightly outperforming the 25 weighted one at the most stringent cutoffs but underperforming at laxer thresholds. The best subsignature performance for each metric was as follows: top10 NDCG of 0.0540 (log weighted), overall NDCG of 0.191 (log weighted), partial AUROC of 0.00821 (unweighted), and overall AUROC of 0.685 (25 weighted). The all similar variant of the primary pipeline, which uses the largest possible similarity list cutoff, performed the worst; it had a top10 NDCG of 0.0319, overall NDCG of 0.172, partial AUROC of 0.00637, and overall AUROC of 0.648. Its lower performance compared to the other primary pipeline variants was expected based on the results of our similarity list cutoff optimization trial (section 4.1). This shows some promise for the subsignature pipeline: though it underperforms the mature and optimized primary pipeline, it overperforms a suboptimal implementation of the primary pipeline. Thus, the subsignature pipeline may be able to match or exceed the primary pipeline with further optimization or through the implementation of consensus scoring strategies, which is the major feature lacking in the underperforming all similar pipeline.

This head-to-head assessment of the primary pipeline replicated trends observed using our new internal benchmarking protocol. CANDO performed best when the TTD drug-indication mapping was used on both assessments. Both our head-to-head assessment and our parameter optimization trial showed worse performance at higher (less stringent) similarity list cutoffs past the optimal value (section 4.1). This correspondence of results provides additional validation to the findings of our benchmarking protocols. However, we did not internally benchmark a pipeline like the multiple lists variant, which performed best in our head-to-head assessment. Future work can explore this new pipeline, which may result in further improvements to the consensus scoring protocol of CANDO. This demonstrates yet another benefit of thorough benchmarking: comparison of platforms may inspire refinements that would otherwise be overlooked.

## 5 Concluding remarks

Drug discovery benchmarking should be accurate, output results that are realistic to novel prediction scenarios, and allow comparison between platforms and technologies. We updated the internal benchmarking protocol of the CANDO platform and created a head-to-head protocol in service of these goals. We assessed CANDO using both protocols. CANDO recalled up to 12.1% of approved drugs in the top 10 compounds for their respective indications; this rose to 31.0% for the top 100 compounds. Positive correlations were observed between performance and the number of drugs associated with an indication, the output of our previous benchmarking protocol, and the drug-drug chemical signature similarity within an indication. Finally, we were able to compare a new drug discovery pipeline to the primary pipeline of CANDO using our head-to-head benchmarking protocol, which allows comparison of disparate pipelines, whether similarity-based or not.

We evaluated performance using multiple metrics, both new and old, in this study. nAIA proved to be useful because it can be directly related to practical performance above a certain rank. nNDCG resulted in a smaller range of optimal similarity list cutoff values used for optimization, likely due to its inherent prioritization of top-ranking true positives. This lower volatility makes it an attractive metric for future optimization studies. AUROC is a well known and comprehensive metric; however, all false positive thresholds are equally weighted in AUROC calculations. Using predictions with a false positive rate of even 0.1 in a real scenario could require screening hundreds or thousands of drugs, which is rarely practical. The majority of AUROC therefore comes from thresholds that are not practically useful in drug discovery. This is the reason the 25 weighted subsignature pipeline had a better AUROC than the other subsignature pipelines, despite other metrics indicating it is inferior. NDCG similarly suffers when used without a cutoff: the overall nNDCGs calculated by our internal benchmarking protocols in our final assessment using CTD were over two times the top100 nNDCGs measured using the same similarity list cutoff. This indicates that the majority of the overall nNDCG was based on drugs appearing at ranks that are unlikely to be useful in practical application. Therefore, we recommend that more drug discovery studies report partial AUROC and NDCG with a reasonable cutoff among their primary metrics, consistent with previous recommendations. [15, 92]

We focused on the CANDO platform in this study. That being said, we invite researchers working on drug discovery to compare their platforms head-to-head with CANDO and others using the drug-indication mappings we collated and our head-to-head benchmarking protocol devised for this purpose, which is publicly available via Github at https://github.com/ram-compbio/compbench. Comparison will help develop the field, ensure the reliability of published platforms, and inspire new refinements to the assessed platforms. This work may also serve as a fundamental model of internal benchmarking to be refined, expanded upon, and employed for thorough optimization and assessment of drug discovery platforms and pipelines.

## Supplementary Material

Supplement 1

Supplement 2

Supplement 3

Supplement 4
