# Supplementary material for: Strategies for robust, accurate, and generalizable benchmarking of drug discovery platforms
Source: bioRxiv. 2025 Aug 11:2024.12.10.627863. Originally published 2024 Dec 16. Preprint. [Version 2] doi: 10.1101/2024.12.10.627863 (PMC11702551; doi:10.1101/2024.12.10.627863)
Supplement: Supplement 1 [file media-1.pdf]

Supplementary figure 1 – Indication size

CTD, excluding other indicated compounds from rankings

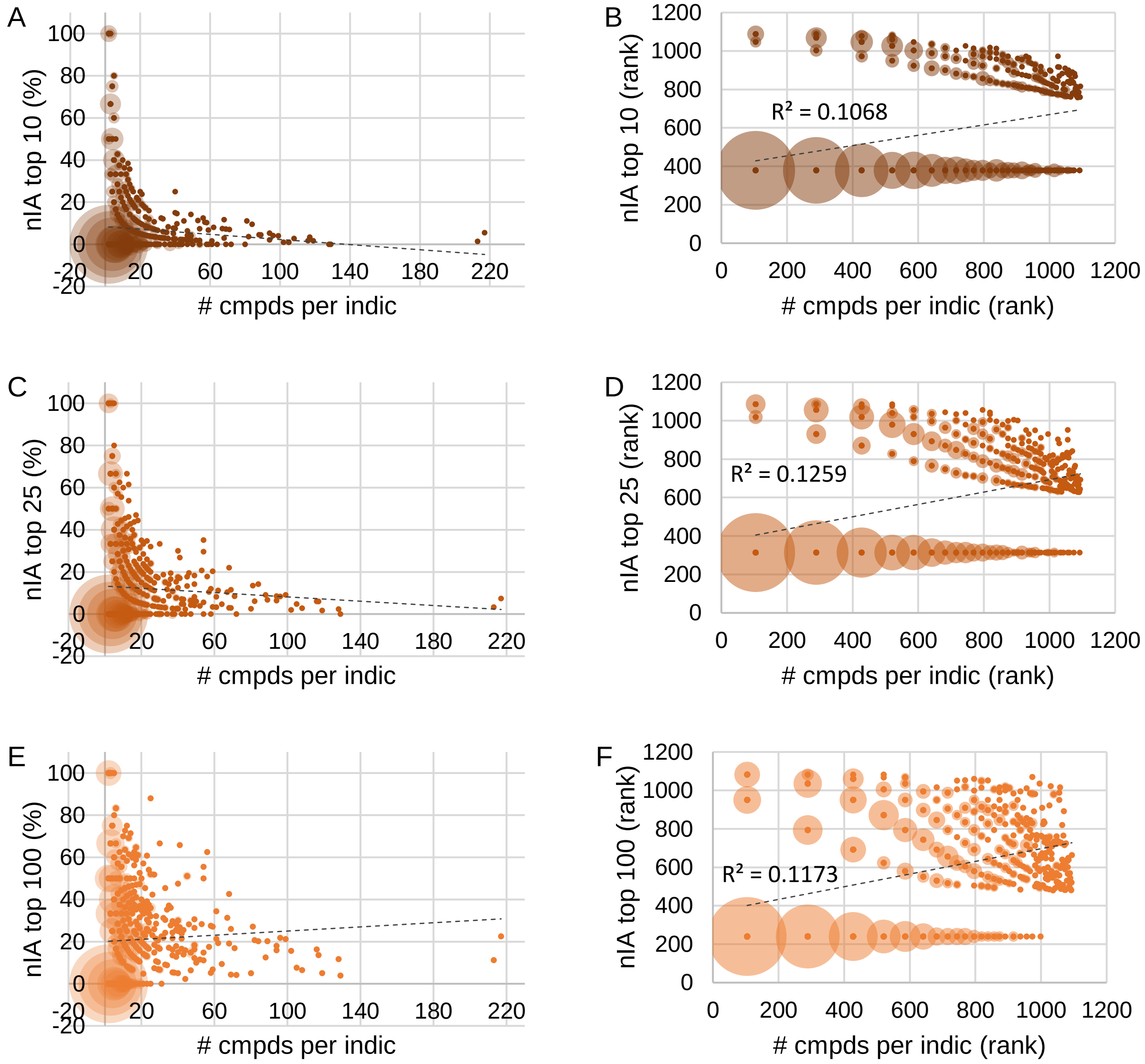

TTD, excluding other indicated compounds from rankings

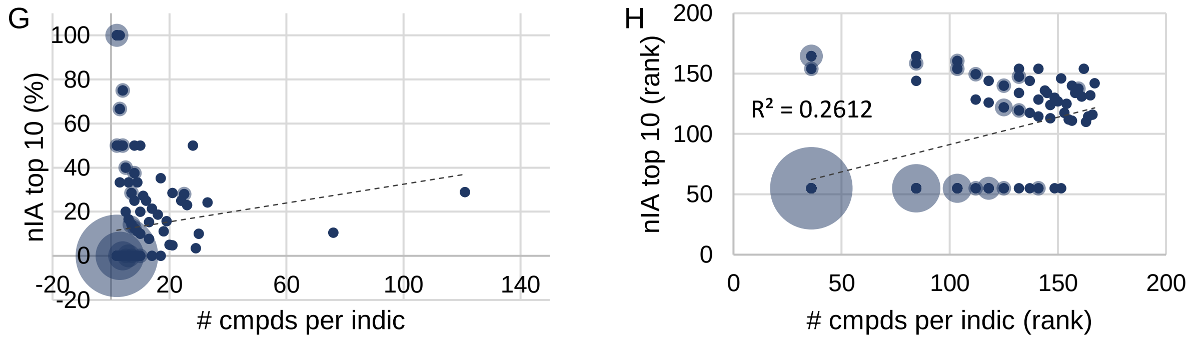

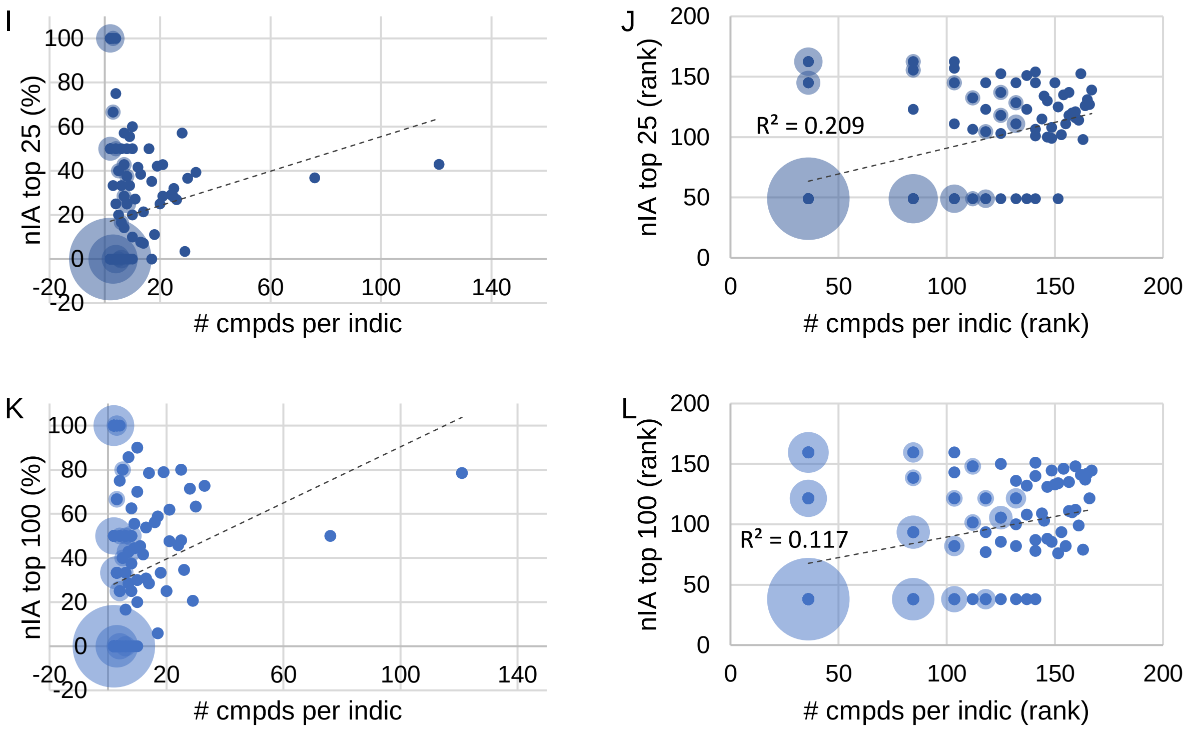

I
nIA top 25 (%)
cmpds per indic
J
nIA top 25 (rank)
# cmpds per indic (rank)
R² = 0.209
K
nIA top 100 (%)
cmpds per indic
L
nIA top 100 (rank)
# cmpds per indic (rank)
R² = 0.117

Supplementary figure 2 – nIA versus IA

CTD

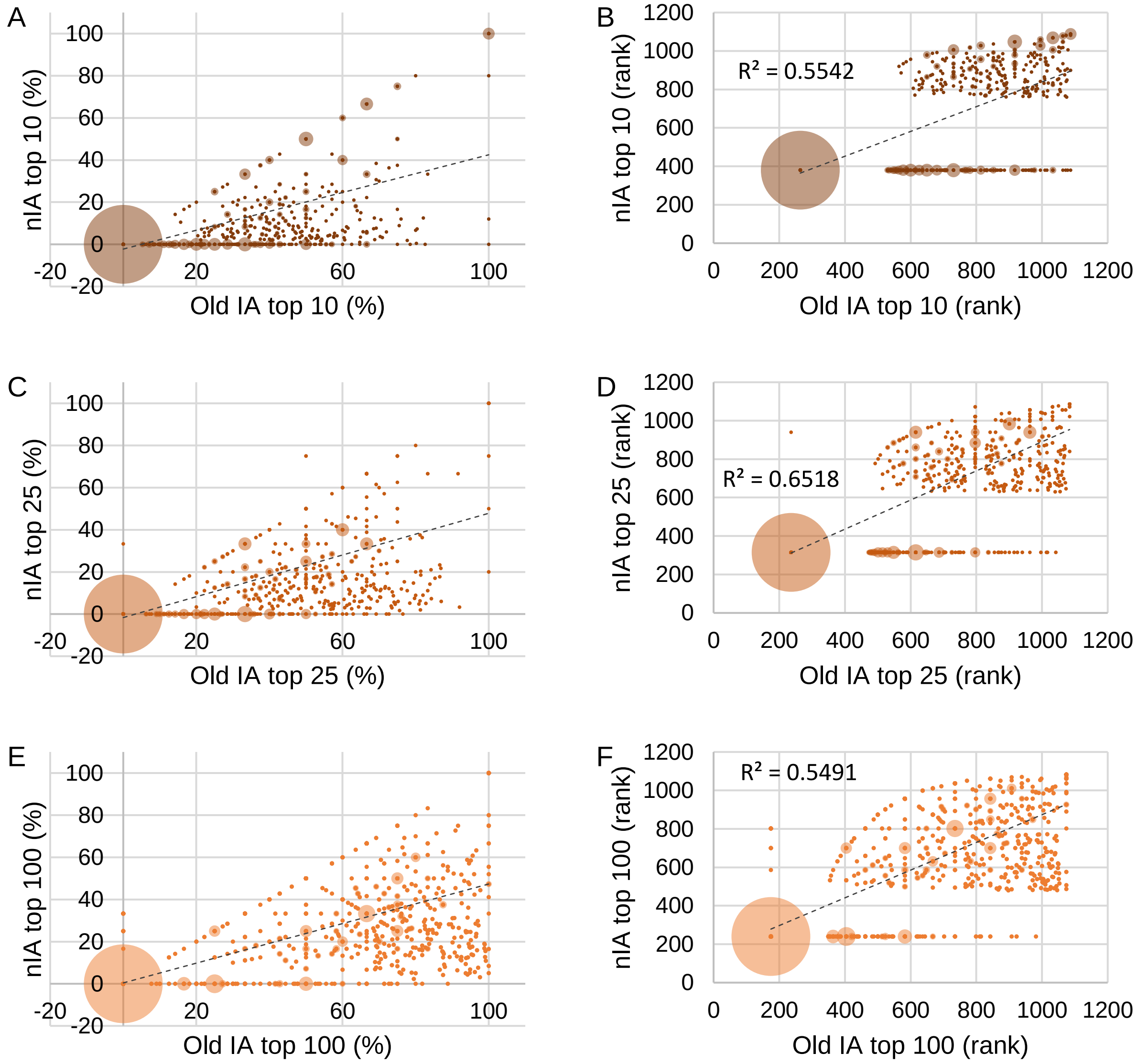

TTD

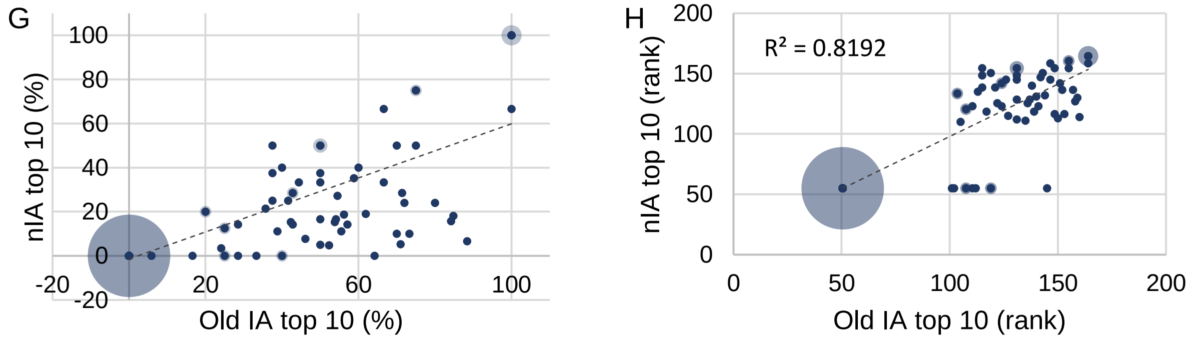

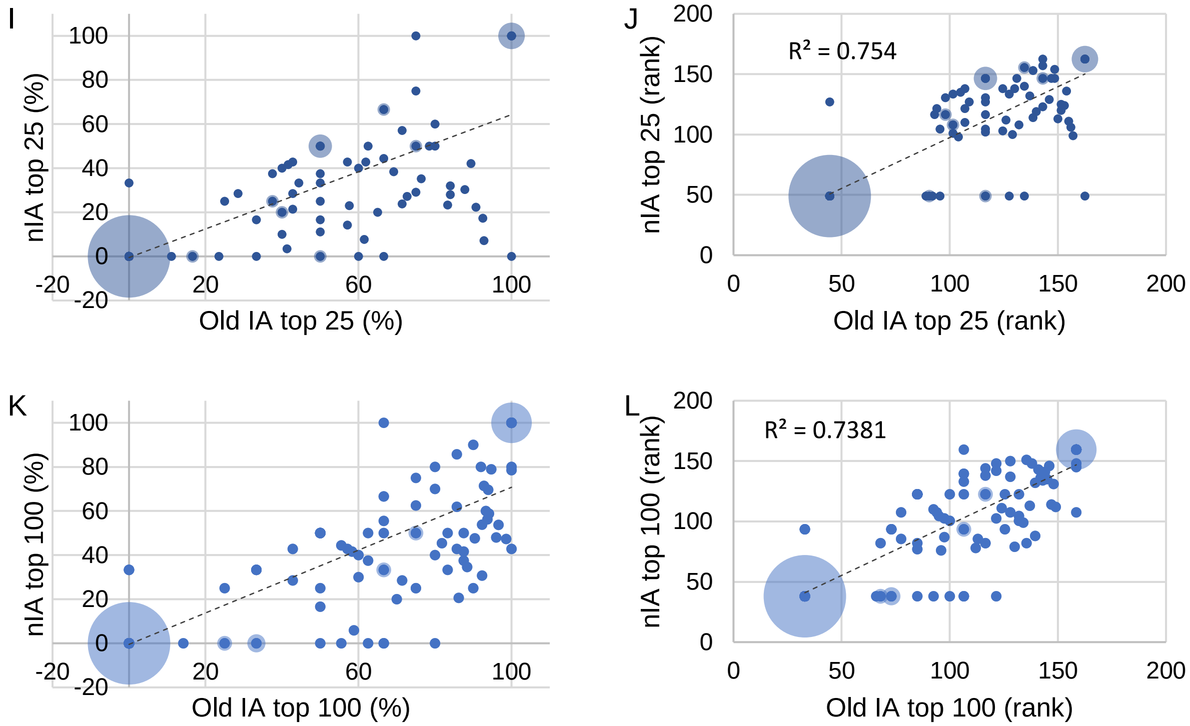

I
nIA top 25 (%)
Old IA top 25 (%)
J
R² = 0.754
nIA top 25 (rank)
Old IA top 25 (rank)
K
nIA top 100 (%)
Old IA top 100 (%)
L
R² = 0.7381
nIA top 100 (rank)
Old IA top 100 (rank)

Supplementary figure 3 – Compound similarity

CTD

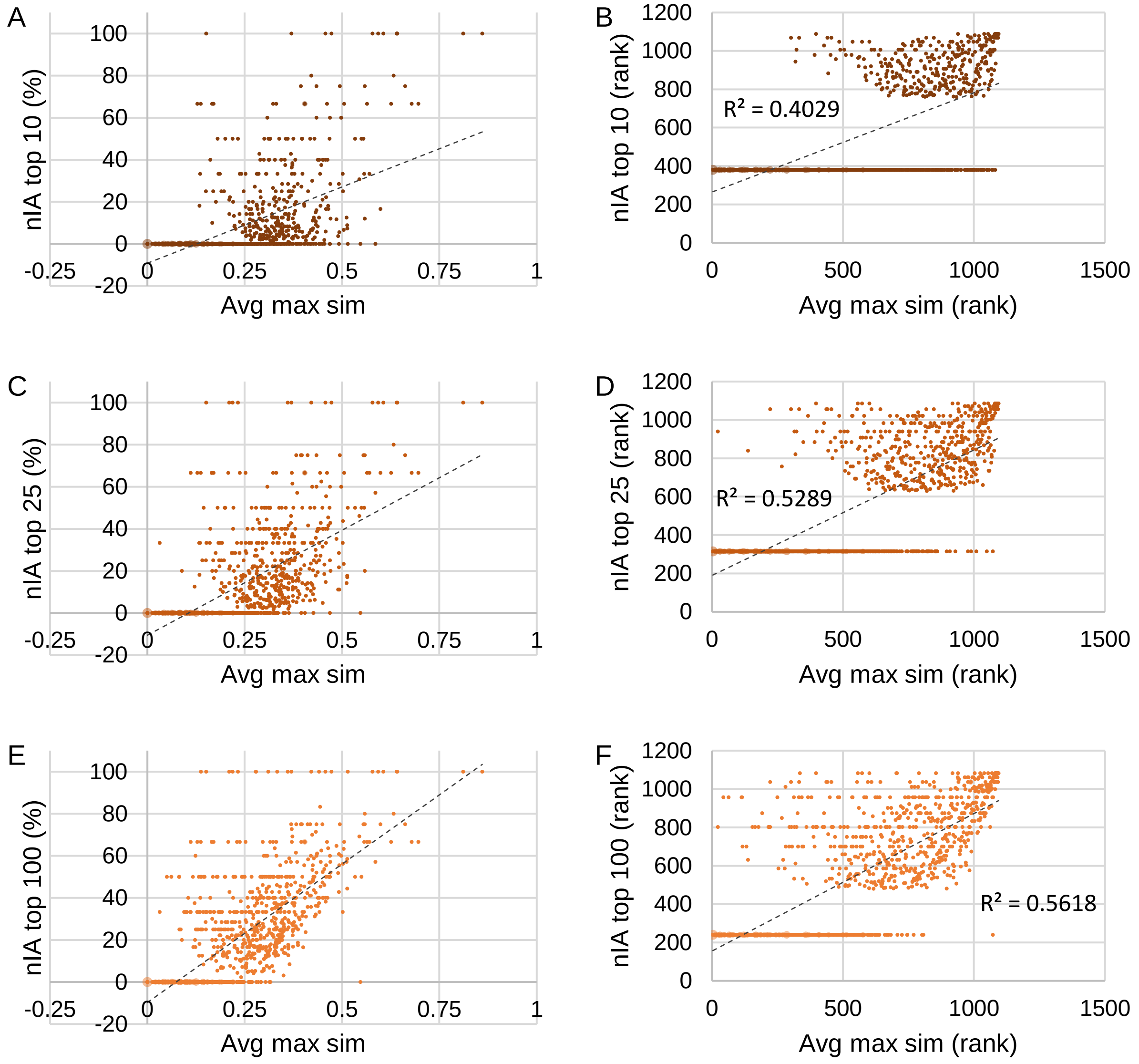

TTD

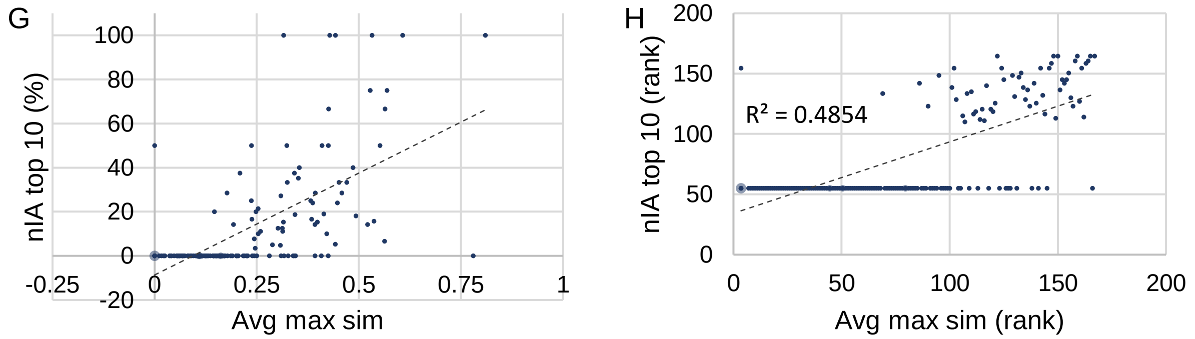

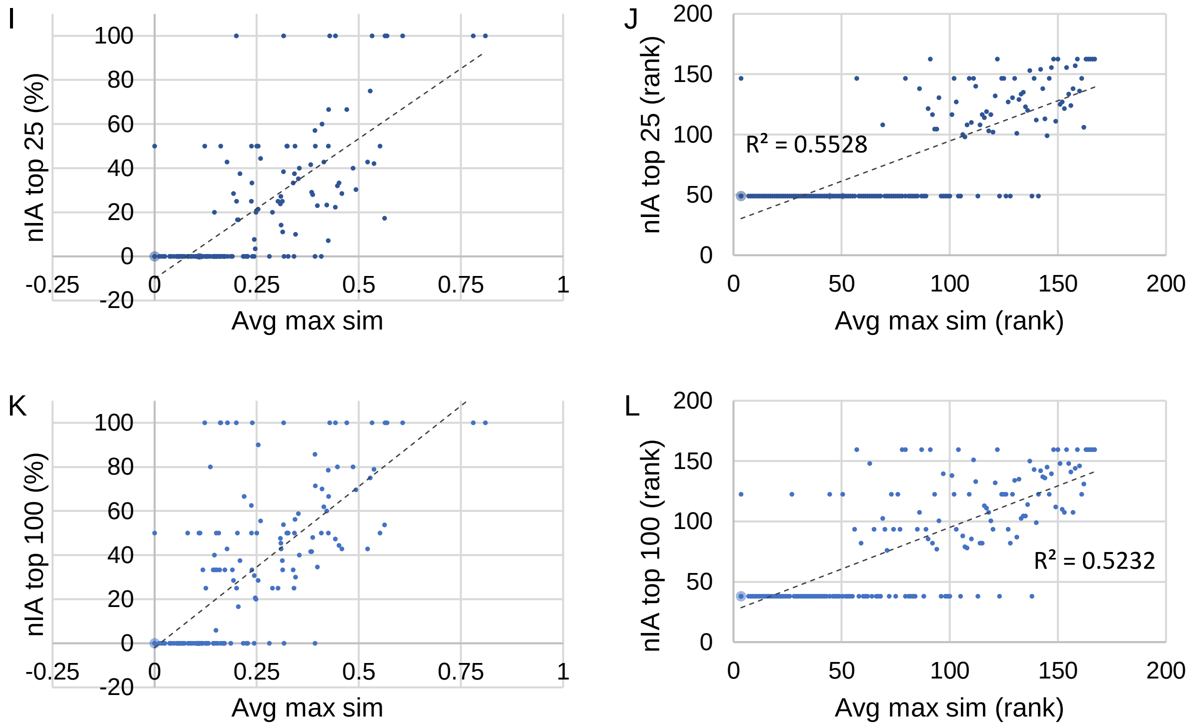

I
nIA top 25 (%)
Avg max sim
J
nIA top 25 (rank)
$R^2 = 0.5528$
Avg max sim (rank)
K
nIA top 100 (%)
Avg max sim
L
nIA top 100 (rank)
$R^2 = 0.5232$
Avg max sim (rank)
